# Neighborhood Disparities in Prevalence of Childhood Obesity Among Low-Income Children Before and After Implementation of New York City Child Care Regulations

**DOI:** 10.5888/pcd11.140152

**Published:** 2014-10-16

**Authors:** Jackson P. Sekhobo, Lynn S. Edmunds, Karen Dalenius, Jan Jernigan, Christopher F. Davis, Mark Giddings, Catherine Lesesne, Laura Kettel Khan

**Affiliations:** Author Affiliations: Lynn S. Edmunds, Mark Giddings, New York State Department of Health, Albany, New York; Karen Dalenius, Jan Jernigan, Centers for Disease Control and Prevention, Atlanta, Georgia; Christopher F. Davis, University at Albany School of Public Health, Albany, New York; Catherine Lesesne, ICF International, Atlanta, Georgia.

## Abstract

**Introduction:**

New York City Article 47 regulations, implemented in 2007, require licensed child care centers to improve the nutrition, physical activity, and television-viewing behaviors of enrolled children. To supplement an evaluation of the Article 47 regulations, we conducted an exploratory ecologic study to examine changes in childhood obesity prevalence among low-income preschool children enrolled in the Nutrition Program for Women, Infants, and Children (WIC) in New York City neighborhoods with or without a district public health office. We conducted the study 3 years before (from 2004 through 2006) and after (from 2008 through 2010) the implementation of the regulations in 2007.

**Methods:**

We used an ecologic, time-trend analysis to compare 3-year cumulative obesity prevalence among WIC-enrolled preschool children during 2004 to 2006 and 2008 to 2010. Outcome data were obtained from the New York State component of the Centers for Disease Control and Prevention’s Pediatric Nutrition Surveillance System.

**Results:**

Early childhood obesity prevalence declined in all study neighborhoods from 2004–2006 to 2008–2010. The greatest decline occurred in Manhattan high-risk neighborhoods where obesity prevalence decreased from 18.6% in 2004–2006 to 15.3% in 2008–2010. The results showed a narrowing of the gap in obesity prevalence between high-risk and low-risk neighborhoods in Manhattan and the Bronx, but not in Brooklyn.

**Conclusion:**

The reductions in early childhood obesity prevalence in some high-risk and low-risk neighborhoods in New York City suggest that progress was made in reducing health disparities during the years just before and after implementation of the 2007 regulations. Future research should consider the built environment and markers of differential exposure to known interventions and policies related to childhood obesity prevention.

## Introduction

Following decades of rising prevalence of obesity among children in the United States, evidence suggests that the trend may be subsiding (1–4). Although childhood obesity has begun to stabilize in New York City, disparities in the burden of obesity and related chronic disease persist (4,5). The causes of childhood obesity are complex; therefore, for prevention efforts to succeed, strategies need to be implemented at multiple levels involving both environmental and policy changes (6,7). Furthermore, involvement at the local public health level is necessary for programs and policies to have population-wide impact (8).

As part of New York City’s efforts to promote health equity and reduce neighborhood health disparities, the New York City Department of Health and Mental Hygiene established a District Public Health Office (DPHO) in the neighborhood that had the highest rates of illness and death in 2002 in each of 3 New York City boroughs: Manhattan, Brooklyn, and the Bronx. DPHOs work with community partners to plan and implement health promotion initiatives in the catchment areas of their respective high-risk neighborhoods. To prevent childhood obesity, personnel in child care centers in DPHO catchment areas receive additional training and technical assistance for promoting physical activity and healthy nutrition than do child care centers in non-DPHO neighborhoods. The additional training and technical assistance provided by DPHOs would be expected to enhance the implementation of various initiatives to prevent childhood obesity in the 3 DPHO high-risk neighborhoods. Key childhood obesity prevention initiatives implemented after the DPHOs were established include the 2006 revisions to Article 47 of the New York City Health Code and the Eat Well Play Hard in Child Care Settings initiative, which was launched by the New York State Department of Health at child care centers participating in the federally funded Child and Adult Care Food Program. Both initiatives sought to improve the nutrition, physical activity, and television-viewing behaviors of children enrolled in licensed child care centers.

Many children, especially low-income children, attend nonlicensed child care or have home day care providers; these children are also likely to be enrolled in public health nutrition programs such as WIC. Indeed, more than half (52.0%) of children sampled from child care centers in low-income neighborhoods for the 2-part ICF International (ICF) evaluation of New York City Article 47 child care regulations, which is described elsewhere (9) in this issue of *Preventing Chronic Disease,* were also enrolled in WIC (9). No population-wide data source is available at the local level for monitoring changes in obesity among preschool-aged children. Data from WIC are a reliable source of information on measured weight and height of enrolled low-income, preschool children (10). Accordingly, to supplement the ICF evaluation, we used New York State WIC data from the Centers for Disease Control and Prevention’s (CDC’s) Pediatric Nutrition Surveillance System (PedNSS) 1) to conduct an exploratory ecologic study of changes in obesity prevalence and 2) to compare disparities in obesity prevalence between preschool children enrolled in WIC in DPHO (ie, high-risk) areas and preschool children in non-DPHO (ie, low-risk) neighborhoods 3 years before (2004–2006) and 3 years after (2008–2010) the initial response to the new child care regulations in 2007.

## Methods

We used an ecologic, time-trend analysis to compare trends in early childhood obesity prevalence between 2004–2006 and 2008–2010 in New York City high-risk neighborhoods located in and around DPHO areas (11). Child care centers in the DPHO neighborhoods were oversampled in the ICF evaluation to study compliance with the regulations by child care centers in predominantly low-income neighborhoods. Data for calculating obesity prevalence and racial/ethnic distributions were obtained from the New York State component of CDC’s PedNSS, for 2004–2006 (n = 148,785) and 2008–2010 (n = 170,091).

The CDC PedNSS monitored height and weight of all preschool children enrolled in WIC in New York State during the study time frame. Clinic- and county-specific data were captured to assess obesity trends in the New York City pediatric WIC population. On average, children were assessed twice a year by the WIC program; to measure height and weight, trained staff used a standard protocol (3) or obtained the data from medical referral records. Each child’s age, sex, race/ethnicity, household size, and income were reported by the child's parent or caregiver. For this study, race/ethnicity was categorized as non-Hispanic black, non-Hispanic white, Hispanic, or other; the “other” group is small and consists of numerous racial/ethnic categories, including South Asian, East Asian, Native American, and Pacific Islander. Household income was converted to a ratio of income-to-federal-poverty level based on household size by using annual federal poverty guidelines for 2004 or 2010 (12). Data were collected at the clinic level, aggregated at the state level, and submitted to CDC for analysis. Weight, height, and age data were used to calculate body mass index (BMI) (weight [kg]/height [m^2^]). For children aged 2 to younger than 5 years, obesity is defined as BMI for age at or below the 95th percentile on the basis of the 2000 CDC sex-specific growth charts (13). One record per child per year was randomly selected to estimate annual obesity prevalence. Weight and height data were excluded if data were missing, miscoded, or biologically implausible (13,14).

Only New York City PedNSS records for children aged from 3 through 4 years were included in the study sample to ensure comparability with preschool-aged children included in the New York City child care evaluation. Data for 2007 were excluded because that was the year the New York City child care regulations were implemented.

To maximize the comparability of New York State PedNSS data with data from child care centers included in the New York City evaluation, WIC clinics in the 5 boroughs were mapped with ArcView, Version 10.0 (Environmental Systems Research Institute, Inc) against child care centers included in the evaluation and compared visually. Because of the relatively small number of child care centers in the boroughs of Queens and Staten Island included in the New York City evaluation, our analysis was restricted to study centers in and surrounding the DPHO areas of the Bronx, Brooklyn, and Manhattan boroughs, hereafter referred to as “high-risk neighborhoods.” WIC clinics located outside the DPHO-catchment areas and in the rest of each borough constituted the “low-risk neighborhoods.” These study areas were defined to be consistent with definitions used for New York City’s efforts to reduce health disparities in areas that were deemed to be high-need.

To assess possible demographic shifts in the WIC-enrolled children across the study areas, 2004 and 2010 New York City PedNSS racial/ethnic distributions were compared by using absolute percentage changes. For the purposes of this study, race/ethnicity was used as a marker of differential exposure to obesogenic social factors (15). We did not standardize obesity prevalence for race/ethnicity because we did not have estimates of obesity prevalence by race/ethnicity for individual New York City WIC clinics. Data on the household income of WIC-enrolled children were used to calculate the mean poverty ratio in 2004 and 2010 for each of the borough-specific high-risk neighborhoods and their corresponding low-risk neighborhoods. Similarly, 3-year obesity prevalence estimates before 2007 (2004–2006) and after 2007 (2008–2010) were computed for borough-specific high-risk and low-risk neighborhoods, and the significance of changes in 3-year obesity prevalence was assessed by using χ^2^ tests. Trends in childhood obesity prevalence were assessed by using a log-linear model in PROC REG, SAS version 9.3 (SAS Institute, Inc).

## Results

The figure  displays the spatial distribution of WIC clinics included in our study along with child care centers targeted for the ICF evaluation of Article 47 regulations. The figure also shows the high-risk neighborhoods served by a DPHO and shows adjacent low-risk neighborhoods located outside the DPHO catchment areas in the boroughs of Manhattan, Brooklyn, and the Bronx. In addition to showing the target areas for DPHO activities and initiatives (ie, high-risk neighborhoods), the map also shows the clustering of WIC sites and low-income child care centers across all 3 study areas. The high-risk study areas include 46 of the 86 WIC clinics in New York City (53%). In 2004, 84% of WIC participants in the high-risk neighborhoods in our study area (n = 32,710) were Hispanic (52%) or non-Hispanic black (32.0%). The proportions of the 2 subgroups changed little in the high-risk neighborhoods in 2010. In contrast, in the low-risk areas, non-Hispanic black and Hispanic children together constituted approximately half of WIC-enrolled children in 2004 (51%) and 2010 (49%). Among high-risk neighborhoods, the largest absolute change in racial/ethnic composition from 2004 through 2010 occurred in the Bronx where the proportion of WIC-enrolled Hispanic children increased by 4.9 percentage points, while the proportion of children in the “other” category decreased by 5.4 percentage points (Table 1). A slight increase in the proportion of non-Hispanic black children was observed in the Manhattan high-risk neighborhoods (2.2%) along with a small decline in the proportion of children in the “other” category (2.9%). Among the low-risk neighborhoods, the largest changes in racial/ethnic composition occurred in Brooklyn where the proportions of WIC-enrolled non-Hispanic white and non-Hispanic black children both decreased by 4.8% percentage points while the proportion of children in the “other” category increased by 6.3%. In both 2004 and 2010, the household income of WIC-enrolled children in high-risk neighborhoods tended to be a lower proportion of the federal poverty level than that of their WIC-enrolled counterparts in low-risk neighborhoods (Table 1). On average, WIC-enrolled children in high-risk neighborhoods in 2004 lived in households with incomes at approximately 79% of the federal poverty level compared with 88% of those in low-risk neighborhoods. This pattern was unchanged in 2010. Comparisons of the mean poverty ratio between 2004 and 2010 in both high- and low-risk neighborhoods showed a general decline, with the largest decreases observed in Brooklyn (high-risk neighborhoods, −0.29; low-risk neighborhoods, −0.24) and the smallest in the Bronx (high-risk neighborhoods, −0.13; low-risk neighborhoods,−0.04).

**Figure:**
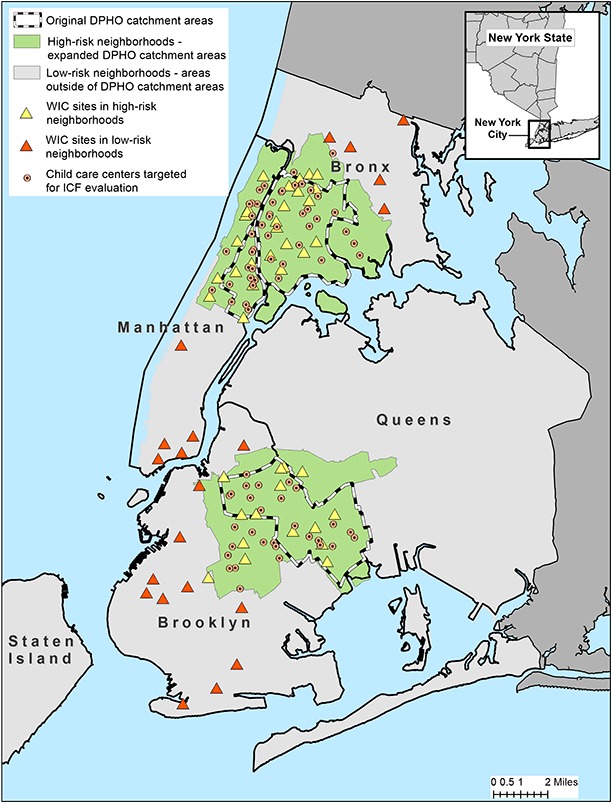
Child care centers and Special Supplemental Nutrition Program for Women, Infants, and Children (WIC) clinics in high-risk and low-risk neighborhoods of Manhattan, Brooklyn, and the Bronx. Solid and dotted lines indicate the boundaries of the New York City District Public Health Offices (DPHO) located in Central and East Harlem in Manhattan, North and Central Brooklyn, and the South Bronx.

**Table 1:** Characteristics of Children Aged 3 to 4 Years (N = 110,773) Residing in High- and Low-Risk Neighborhoods in Manhattan, Brooklyn, and the Bronx and Enrolled in the Special Supplemental Nutrition Program for Women, Infants, and Children, New York City, 2004–2010

Characteristic	High-Risk Neighborhoods^a^			Low-Risk Neighborhoods^b^				
	2004 n = 34,079	2010 n = 40,701	Difference^c^	*P* Value	2004 n = 15,536	2010 n = 20,457	Difference^c^	*P* Value
	n (%)	n (%)			n (%)	n (%)		
**The Bronx**								
Non-Hispanic black	2,814 (24.0)	3,643 (24.9)	0.9	<.001	1,255 (36.3)	1,719 (35.4)	−0.9	.051
Non-Hispanic white	146 (1.2)	138 (0.9)	−0.3		136 (3.9)	207 (4.3)	0.4	
Hispanic	7,702 (65.8)	10,367 (70.7)	4.9		1,674 (48.4)	2,282 (47.0)	−1.4	
Other	1,043 (8.9)	510 (3.5)	−5.4		395 (11.4)	648 (13.3)	1.9	
Poverty ratio^d^, mean (SD)	0.74 (0.52)	0.61 (0.45)	−0.1	<.001	0.81 (0.56)	0.77 (0.55)	−0.04	.004
**Brooklyn**												
Non-Hispanic black	6,388 (45.7)	7,517 (43.9)	−1.8	.059	1,550 (15.0)	1,586 (10.2)	−4.8	<.001
Non-Hispanic white	2,924 (20.9)	3,509 (20.5)	−0.4		4,131 (39.9)	5,462 (35.1)	−4.8	
Hispanic	3,817 (27.3)	4,696 (29.0)	1.7		2,799 (27.0)	3,880 (24.9)	−2.1	
Other	836 (6.0)	1,111 (6.5)	0.5		1,867 (18.0)	3,499 (24.3)	6.3	
Poverty ratio^d^, mean (SD)	0.90 (0.50)	0.61 (0.43)	−0.29	<.001	0.93 (0.47)	0.69 (0.40)	−0.24	<.001
**Manhattan**												
Non-Hispanic black	1,344 (19.0)	1,517 (21.2)	2.2	<.001	225 (7.3)	251 (8.5)	1.2	.008
Non-Hispanic white	39 (0.6)	143 (2.0)	1.4		27 (0.9)	54 (1.8)	0.9	
Hispanic	5,324 (75.4)	5,350 (74.7)	−0.7		1,129 (36.7)	1,091 (37.0)	0.3	
Other	353 (5.0)	149 (2.1)	−2.9		1,696 (55.1)	1,556 (52.7)	−2.4	
Poverty ratio^d^, mean (SD)	0.74 (0.49)	0.63 (0.44)	−0.11	<.001	0.87 (0.50)	0.69 (0.40)	−0.18	<.001

Abbreviation: SD, standard deviation.

a High-risk neighborhoods are the areas in or adjacent to the District Public Health Office areas of the Bronx, Brooklyn, and Manhattan boroughs.

b Low-risk neighborhoods are the areas in each borough outside the District Public Health Office catchment area.

c Values are percentages unless otherwise indicated. Percentages may not total 100 because of rounding.

d Poverty ratio is ratio of income to federal poverty level computed by income and household size using annual the US Department of Health and Human Services’s Federal Poverty Guidelines for 2004 (24) or 2010 (25).

The 3-year prevalence of childhood obesity among 3- and 4-year old children enrolled in WIC during 2004–2006 and 2008–2010 was consistently higher in high-risk neighborhoods than in low-risk neighborhoods (Table 2). The highest prevalence among both high-risk and low-risk neighborhoods was in the Bronx. Childhood obesity prevalence declined in all study neighborhoods. Changes were significant in all areas except in Manhattan low-risk neighborhoods. The greatest decline occurred in Manhattan high-risk neighborhoods where childhood obesity prevalence decreased from 18.6% during 2004–2006 to 15.3% during 2008–2010 (*P* < .001). This decline led to a substantial narrowing of the Manhattan gap in childhood obesity prevalence between high-risk and low-risk neighborhoods. In the Bronx, childhood obesity prevalence in high-risk neighborhoods declined from 19.1% in 2004–2006 to 17.1% in 2008–2010 (*P* < .001) and reached parity with that of the Bronx low-risk neighborhood during 2004–2006 (17.4%) leading to a slight narrowing of the gap. A similar narrowing of the gap was not observed in Brooklyn.

**Table 2:** Prevalence of Obesity Among 3- and 4-Year-Old Children Enrolled in the Special Supplemental Nutrition Program for Women, Infants, and Children in High-Risk Versus Low-Risk Study Neighborhoods in Manhattan, Brooklyn, or the Bronx Before (2004–2006) and After (2008–2010) Implementation of New York City Day Care Policies.

Borough	High-Risk Neighborhoods			*P* Value	Low-Risk Neighborhoods			*P* Value
	2004–2006	2008–2010	Change		2004–2006	2008–2010	Change	
Bronx	19.1	17.1	–2.0	<.001	17.4	16.1	–1.3	.008
Brooklyn	15.7	14.8	–0.9	<.001	13.6	12.8	–0.8	.004
Manhattan	18.6	15.3	–3.3	<.001	12.0	11.5	–0.5	.302

The average annual percentage change in prevalence of obesity in high-risk neighborhoods from 2004 through 2010 was −2.6% (*P* = .007) compared with −1.6% (*P* = .082) in low-risk neighborhoods. The highest annual percentage change occurred in the Manhattan high-risk neighborhood (−4.7%; *P* < .001), followed by that in the Bronx high-risk neighborhood (−2.6%, *P* = .005). No significant trends were observed in the Brooklyn high-risk neighborhood or in any of the 3 low-risk neighborhoods (data not shown).

## Discussion

The results of this study show that 3-year obesity prevalence among 3- and 4-year old children enrolled in WIC in high-risk and low-risk neighborhoods in the Bronx, Brooklyn, and Manhattan declined from 2004–2006 to 2008–2010. The declines were greatest in high-risk neighborhoods of the Bronx and Manhattan, with average annual percentage changes that ranged from −4.7% in Manhattan to −2.6% in the Bronx. The findings suggest a narrowing of the gap in early childhood obesity prevalence between high-risk and low-risk neighborhoods in Manhattan and the Bronx, but not in Brooklyn where race/ethnicity shifts in low-risk neighborhoods were substantial. The observed narrowing of the gap between 3-year obesity prevalence in high-risk neighborhoods in Manhattan and the Bronx during 2008–2010 suggests that some progress is being made in addressing health disparities consistent with the mission of the New York City DPHOs.

The observed declines in 3-year obesity prevalence in the study neighborhoods from 2004–2006 to 2008–2010 are consistent with secular trends that show that obesity among preschool and school-aged children has plateaued (1,2), and with reports of declines in childhood obesity in different parts of the United States (3,4,16,17). The relationship of these findings to compliance with the Article 47 regulations by child care centers in the ICF New York City evaluation is unknown but may point to the importance of more intensive assistance in these areas of the city. Compliance with Article 47 of the New York City Health Code was expected in all city neighborhoods. It is likely that multiple factors influenced the decline in obesity rates.

This study shows that geographic variation in childhood obesity is significant within New York City neighborhoods. Evidence of neighborhood-level variation in childhood obesity prevalence in a large city such as New York City underscores the importance of identifying and monitoring modifiable aspects of the built and social environments when designing and implementing interventions and policies to support the maintenance of healthy lifestyles (18).

Because this study did not include measures of the built environment, it is not possible to comment on the extent to which the observed differences in 3-year obesity prevalence over time could be explained by within-neighborhood changes in socio-environmental characteristics. However, a previous analysis of census and New York City Community Health Survey data shows that more affluent neighborhoods in New York City tend to have more resources that support maintenance of physical activity and healthy eating behavior (18). Most importantly, that same analysis showed that prevalence of adult obesity was higher in less-resourced, low-income communities than in more affluent neighborhoods (18).

Beyond the built environment, however, possible explanations for the observed differences in childhood obesity prevalence among the study neighborhoods range from sociodemographic characteristics of the populations enrolled in WIC to differences in implementation of population-wide obesity prevention policies. In this study, racial/ethnic composition and income-to-poverty ratio were used to assess changes in the sociodemographic characteristics of the study neighborhoods. If the observed declines in 3-year obesity prevalence were largely explained by changes in the racial/ethnic composition of the study neighborhoods, it would be reasonable to expect that proportions of Hispanic and non-Hispanic black children, who are known to be at higher risk for obesity than non-Hispanic white children in the United States (1,3,4,19), would be significantly lower in 2010 than in 2004. However, a careful review of the racial/ethnic composition data shows that the proportions of these 2 high-risk groups remained fairly stable from 2004 to 2010 across all study neighborhoods. Furthermore, in 2010 WIC-enrolled children in each borough were living with families in greater poverty than were the WIC-enrolled children in 2004 as evidenced by the average decrease in income-to-poverty ratio in each borough. This finding suggests that the observed differences in changes in obesity prevalence cannot be attributed to the changing racial/ethnic or socioeconomic composition of the neighborhoods.

In light of the numerous interventions implemented in New York City during the last decade (4) to address the growing childhood obesity epidemic, including the implementation of child care regulations in Article 47 of the New York City Health Code in 2007, the results of this study suggest that citywide policies may be working in concert with state and local initiatives to change the food and physical activity environments for low-income, preschool children. Because data for this study came from WIC-enrolled, low-income children, the observed geographic variation in childhood obesity prevalence trends also raises the possibility that families with WIC-enrolled children are better able to adopt and maintain some healthy lifestyles promoted by the WIC program, such as healthy eating, physical activity, and reduced screen time (20) in some neighborhoods than in others (21).

This study examined childhood obesity prevalence trends at a sub-city level over time. Previous studies of preschool-aged children compared trends at the state (2) or city (19,22) level but not at the neighborhood level. The use of 3-year prevalence proportions instead of annual prevalence proportions ensured that comparisons were made by using more stable numerators (ie, counts of WIC-enrolled obese 3- and 4-year old children) and denominators (ie, counts of all 3- and 4-year-old children enrolled in WIC) across all study neighborhoods during the 2004–2006 and 2008–2010.

Our study has several limitations. Like all ecologic study designs, the findings of this hypothesis-generating study cannot be used to draw causal inferences at the individual level. Second, we had no information on the national origin or length of time in the United States of WIC participants; therefore, it was not possible to assess whether changes in the makeup of racial/ethnic subpopulations contributed to changes in obesity prevalence across the study areas. Furthermore, we cannot rule out the effect of more children who are not at risk of obesity enrolling in WIC as a result of the economic downturn that occurred in New York City and nationwide during 2008–2010. Finally, the scope and reach of health promotion activities in DPHO areas are known to have expanded during the study period; however, we were not able to assess the extent to which this expansion may vary across the DPHO areas or may explain the observed neighborhood disparities in childhood obesity prevalence trends.

Although work still remains to eliminate these health disparities (particularly in the South Bronx where obesity prevalence during both 2004–2006 and 2008–2010 was approximately twice the *Healthy People 2020* (23) target of 9.6% for early childhood), evidence of declines in childhood obesity among children enrolled in WIC in all study areas and a narrowing of the gap between high-risk and low-risk neighborhoods in Manhattan and the Bronx is encouraging. New York State and New York City have been proactive and innovative in childhood obesity prevention with statewide and citywide initiatives focused on improving age-appropriate physical activity and access to affordable healthy foods in early child care and WIC settings (4,22). Future research should include measures of the built environment and individual exposure to known interventions and policies to prevent childhood obesity, including exposure to child care, and should incorporate multilevel regression modeling to fully understand factors associated with childhood obesity prevalence trends in New York City neighborhoods.
